# Connecting physical and social dimensions of place attachment: What can we learn from attachment to urban recreational spaces?

**DOI:** 10.1007/s10901-016-9495-4

**Published:** 2016-01-27

**Authors:** Rebecca Madgin, Lisa Bradley, Annette Hastings

**Affiliations:** Urban Studies, School of Social and Political Sciences, University of Glasgow, Glasgow, UK

**Keywords:** Neighbourhoods, Place attachment, Physical, Sporting recreational spaces, Social bonds, Urban

## Abstract

This paper is concerned with the ways in which people form attachments to recreational spaces. More specifically it examines the relationship between recreational spaces associated with sporting activity in urban neighbourhoods and place attachment. The focus is on the ways in which changes to these spaces exposes the affective bonds between people and their surroundings. The paper applies a qualitative methodology, namely focus groups and photo elicitation, to the case study of Parkhead, a neighbourhood in the East End of Glasgow. Parkhead has historically been subjected to successive waves of redevelopment as a result of deindustrialization in the late twentieth century. More recently redevelopment associated with the 2014 Commonwealth Games involved further changes to neighbourhood recreational spaces, including refurbishing of existing sports facilities and building new ones. This paper reflects on the cumulative impacts of this redevelopment to conclude (a) that recreational sports spaces provoke multi-layered and complex attachments that are inextricably connected to both temporal and spatial narratives and (b) that research on neighbourhood recreational spaces can develop our understanding of the intricate relationship between the social and physical dimensions of place attachment.

## Introduction

Sport and recreation are intricately connected to physical spaces. At the macro level, mega-sporting events are commonly associated with their geographic location. From the Olympic Games in Barcelona 1992 to the Commonwealth Games in Glasgow 2014, the external image of place can become synonymous with sporting reputation. At the micro level, many sports such as football, cricket, and rugby are known as ‘representational’ sports in that they represent places and their histories. As Bale (2000) recognises, almost all football clubs in England are named after places and these sporting traditions are increasingly used to attract human and capital investment (Ramshaw and Gammon 2010). Despite this, there has been very little academic attention paid to the ways in which recreational areas associated with sport contribute to residents’ identification with, and attachment to, urban neighbourhoods. This paper draws on literature from urban studies, environmental psychology, leisure studies and sports history to examine the ways in which such spaces can further our understanding of how people form attachments to urban neighbourhoods.

The political importance of nurturing bonds between people and place is indicated by successive urban policy initiatives designed to foster a sense of place and attachments to place (Scottish Government 2010, 2014). It is also indicated by the long-standing and ever-burgeoning literature on place attachment (Manzo and Devine-Wright 2014; Lewicka 2011). Despite this political and academic interest, we do not yet understand whether and how attachment manifests in relation to certain kinds of spaces within urban neighbourhoods. Thus much of the literature has focused on people’s bonds with residential spaces, rather than with recreational, retail or employment spaces. The aim here is to explore the ways in which people form attachments to recreational spaces, specifically the informal and formal sporting spaces located within an urban neighbourhood. Such spaces include streets, parks or open spaces, local sports centres and major stadia. Common to all is that they are public recreational spaces in which both males and females of any age gather either to play or watch sport. In particular the paper focuses on two main research questions: (1) to what extent are people attached to sporting spaces in their urban neighbourhood, and (2) in what ways are these attachments formed? These questions are explored through a case study of the Parkhead neighbourhood of Glasgow.

The paper is structured as follows. The literature review, which immediately follows, begins with an overview of the place attachment literature in order to examine what is known about attachments to recreational spaces more broadly. It then examines the debate on the social and physical drivers of place attachment. The third section outlines the research design and methodological approach of the project and provides contextual detail on the case study. The fourth section explores the findings of the research and the final concluding section reflects on the significance of these findings for a more holistic understanding of the drivers and manifestations of place attachment within urban areas.

## Place attachment and recreational spaces: evidence from the literature

The term ‘place attachment’ is often used interchangeably with terms such as ‘place identity’ and ‘sense of place’. At the heart of each of these concepts is a desire to understand the relationships that people have with their environment, particularly the emotional aspects of these relationships (Proshansky et al. 1983; Tuan 1977). This paper adopts the position of Altman and Low (1992) to define place attachments as the affective bonds between people and places. Phenomenological studies view ‘place’ as an essential part of our ontological security. For Manzo, place is “an inseparable part of existence” (2003, p. 48). Based on this existential view of place the literature has focused on both the presence of place attachment and the conditions under which these bonds between people and place are formed. Much of the research has been located in particular locations and indeed Lewicka states that around three-quarters of the research studies conducted since the 1970s deal with attachment at the neighbourhood level (2011, p. 212). Within the neighbourhood. ‘home’ has been central to the literature (Manzo 2003, p. 2014). Further studies have attempted to ascertain the influence of scale on place attachment, in particular considering the differences between the city, neighbourhood and home (Lewicka 2010).

By comparison there has been very little research into the spaces where everyday sporting activities of the neighbourhood are conducted. This gap becomes increasingly important in the context of urban redevelopment in which neighbourhood ties to these spaces are repeatedly threatened, often with severe psychological consequences (Fullilove 2004). Power is also important: who can influence the creation, adaptation and destruction of attachment is exposed by urban redevelopment initiatives (Dixon and Durrheim 2000; Hillier and Rooksby 2005). The paper seeks to understand the relationship between sports-orientated recreational spaces and place attachment and, in particular, the ways in which changes to these spaces exposes the affective bonds between people and the spaces.

The study of recreational spaces more broadly conceived is an emerging theme in the place attachment literature. Much of it considers the relationship between particular activities and place attachment and has primarily been concerned with how people relate to natural or out-of-the-city spaces, for example, through activities such as canoeing (Bricker and Kerstetter 2000), or mountaineering (Kyle et al. 2003). More recently researchers working in leisure studies have focused on the role of ‘place’ in sport. This has focused on sports tourism and the ways in which sport can be used to lever investment to an area and attract people (Ramshaw and Hinch 2006; Hinch and Higham 2011). The tension between these as spaces of investment and spaces of attachment is a common theme of the research. Ramshaw and Gammon (2010) for example explore how Twickenham, the English Rugby Union stadium, can be a spiritual ‘home’ for fans as well as a tourist attraction.

Research has also sought to examine the relationship between recreation, community and everyday practices. Light (2013) demonstrated the close connection of living, working and playing in northern England’s rugby league heartlands. Likewise, Stone found in relation to football that the sport was “embedded within the daily routines and spatial practices of people’s lives. For some it is a way of overcoming the monotony of the daily routine, for others it offers the only routine around which their lives can be structured” (2007, p. 175). This was further developed by Robson (2000) and Armstrong (1998) who both examined the relationship between being a supporter of a sports team and the creation and reinforcement of individual and collective identities. As well as positive emotional reactions to sporting places and practices, Bale (2000) identifies negative or conflicted emotions in his examination of the role of the stadium in people’s everyday lives, with proximity not necessarily viewed positively. Much of the literature has focused, therefore, on the role of recreational sporting spaces within people’s daily lives. Their role in relation to place attachment remains an underdeveloped area of research.

Sporting spaces provide an opportunity to examine the ways in which physical aspects of spaces can affect the ways in which people’s attachments to place develop. This runs contrary to the focus of much of the place attachment literature which concentrates on the social drivers of attachment (Lewicka 2011). Livingston et al. (2010) in their study of deprived neighbourhoods found that there was “little evidence of attachment to the physical space or environment with only three respondents making specific reference to these aspects of their neighbourhood” (2010, p. 418). However while Lewicka’s (2011) extensive review suggests a research endeavour dominated by the exploration of social and community relations—or “shared behavioural and cultural processes” (p. 214), she also argues that the extent to which these processes were themselves “the result of perceptual and cognitive processes rooted in physical characteristics of settings” has not been explored (p. 214). Indeed Stedman’s seminal work (2003) is rare in posing the question “is place just a social construction”? Drawing on survey evidence, his study edged the field away from viewing the social and physical as binary opposites as it found that respondents mentioned “environmental features & characteristics of place” almost as often as they did “family and friend related reasons” (34.2 % in comparison with 36.9 %) (2003, p. 673).

Bale (2000) and Wood and Gabie (2011) demonstrated the influence of physical structures on the affective bond between people and place through their work on football stadiums in Scotland and England. Bale’s research on the home ground of Hibernian FC quotes a research participant who stated: “that piece of land is wrapped into my Saturday you know, in the sense that it is consistent with how I conduct Saturday, where I go before the game, who I meet up with, what time I leave” (2000, p. 92). Similarly Wood found that: “football grounds have the power to stir hearts and minds and to evoke and anchor strong visual and social memories” (2011, p. 1199). While sports stadiums inscribe a sense of routine and familiarity and social bonding amongst fans (Stone 2007), the construction, use and type of physical space in which these activities took place can also suggest a symbiotic relationship between the social and physical dimensions of place attachment.

The studies of sport and community referenced above come from leisure studies and sports history. While they do not seek to primarily engage with the place attachment literature, these discussions nonetheless suggest that it is fruitful to bring these literatures together. Furthermore the focus of research on sporting spaces has largely been on high-profile stadiums, rather than on more mundane spaces such as neighbourhood sports centres or informal spaces in which sport takes place in urban neighbourhoods. Whilst we acknowledge that playing and watching sports are different activities we examine them together in this paper in order to analyse the extent to which sporting spaces informs attachment to place. Put simply the physical *spaces* where these encounters take place is the subject of the research rather than just the *type* of activity that takes place within them. Exploring the affective materiality of the range of such spaces—the ways in which people build up meanings, memories and lived experiences—is vital to understanding how the use of recreational spaces stimulates attachments to place. The design of a research study developed to explore this question follows next.

## Research design

The central aim of this research is to understand the ways in which sports-orientated recreational spaces provoke and reveal attachments between people and place. A qualitative approach was adopted to explore the relationship between these spaces and place attachment. Following ethical approval, four focus groups were convened with residents from the case study neighbourhood. The residents were recruited using both an open call and the networks of a key local community organisation that also hosted each focus group.

The groups were convened according to age and level of activity in the neighbourhood. These key elements of the focus groups are outlined in Table 1. In addition there was broadly a gender balance in each group, most residents were white and defined themselves as ‘Scottish’ or ‘British’, and the majority were lifelong Parkhead residents. While the range of participants was not representative of the wide socio-demographic of the case study area in terms of ethnicity, participants did represent a cross section of the long–standing white population of the area. Each focus group was facilitated by one member of the research team, with up to two other members taking notes and observing the behaviours and dynamics of the group. Each lasted for between one and a half and two hours.

**Table 1:** Summary of the focus group participants

Identifier	Composition	Age
FG_Active	Active residents i.e. self-defined on the participant information sheet as ‘actively involved in neighbourhood groups’	20 s–50 s
FG_Mixed	Non-active, mixed residents i.e. self-defined on the participant information sheet as ‘not actively involved in neighbourhood groups’	20 s–50 s
FG_Older	Older persons group	60+
FG_Younger	Younger-persons group	12–16

Recognising that “visual approaches for studying place meanings and attachment have been under-utilised, relative to their potential contribution” (Stedman et al. 2014), this study used photo-elicitation (Collier and Collier 1986) to capture the ways in which changes to recreational spaces within Parkhead provoked an articulation of place attachment. Accordingly, photographs were used to initiate discussion in each focus group. A hard copy set of fifteen photographs taken by the researchers of locations and buildings within the neighbourhood were provided to each group. The set was designed to reflect the multi-faceted nature of the neighbourhood in four categories: amenities (shops, libraries); residential (homes and streets); heritage (older public buildings  and commercial premises.); and leisure (sports facilities, playgrounds).

The focus groups followed a common format so that the introductory section (what are the positive and negative elements of the neighbourhood?) was followed by a four-stage process of photo-elicitation which involved participants being:

1. Invited to select a photograph of a place they felt an attachment to and then explain the reasons for their choice.
2. Asked if there were any locations in the neighbourhood they were attached to for which no photograph had been included and to explain why they felt the photograph should have been included.
3. Reminded of the planned development of the area and asked to choose one photograph which represented an aspect of the built environment they would like to be retained, and one they would wish demolished as part of redevelopment.
4. Shown slides of a further six locations within Parkhead as they currently looked, followed immediately with a digitally altered image showing what redevelopment of the location could look like.

**Fig. 1:**
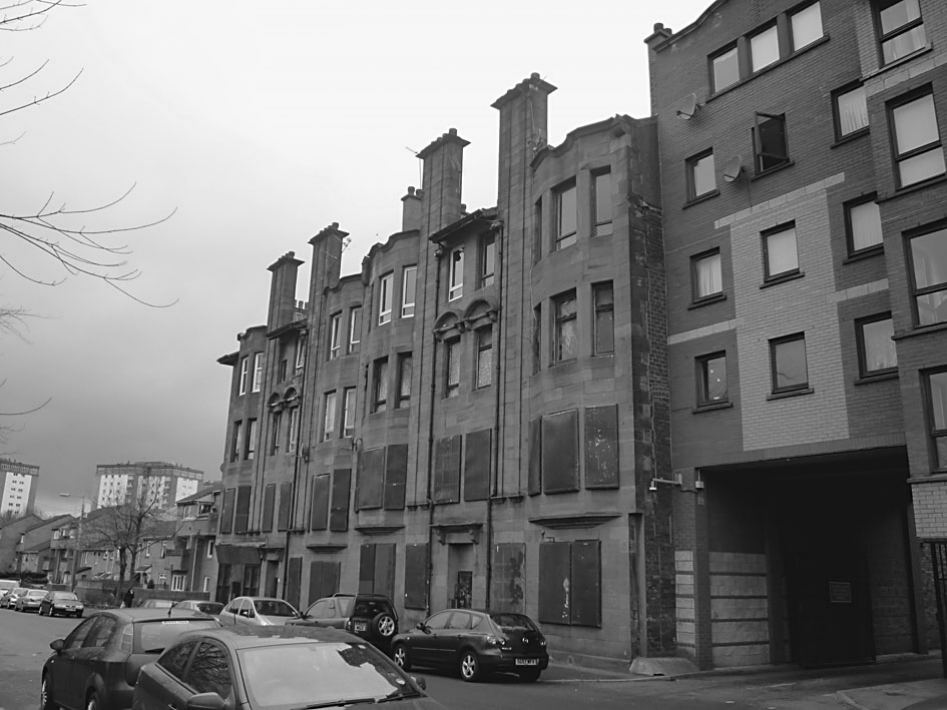
Residential category: different types of housing within Parkhead

**Fig. 2:**
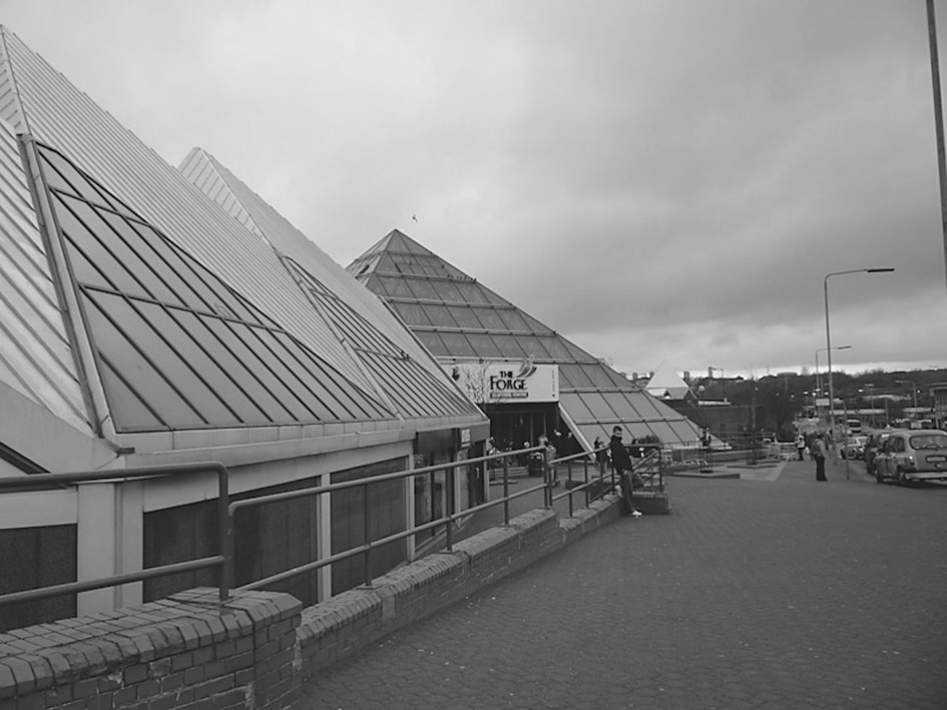
Amenities category: The Forge Shopping Centre

**Fig. 3:**
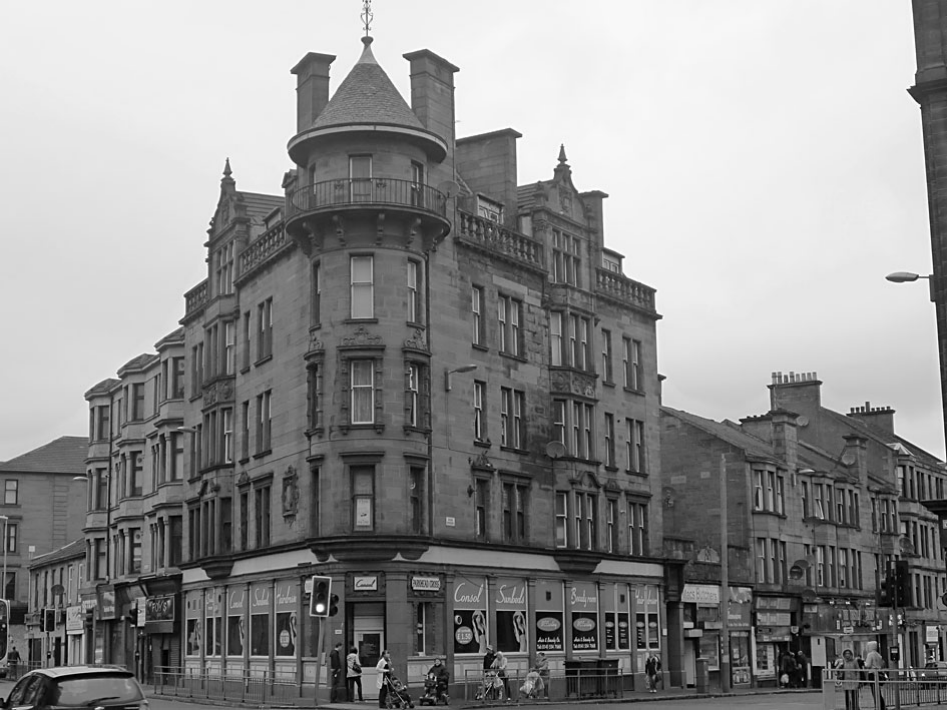
Heritage category: The Historic Parkhead Cross

**Fig. 4:**
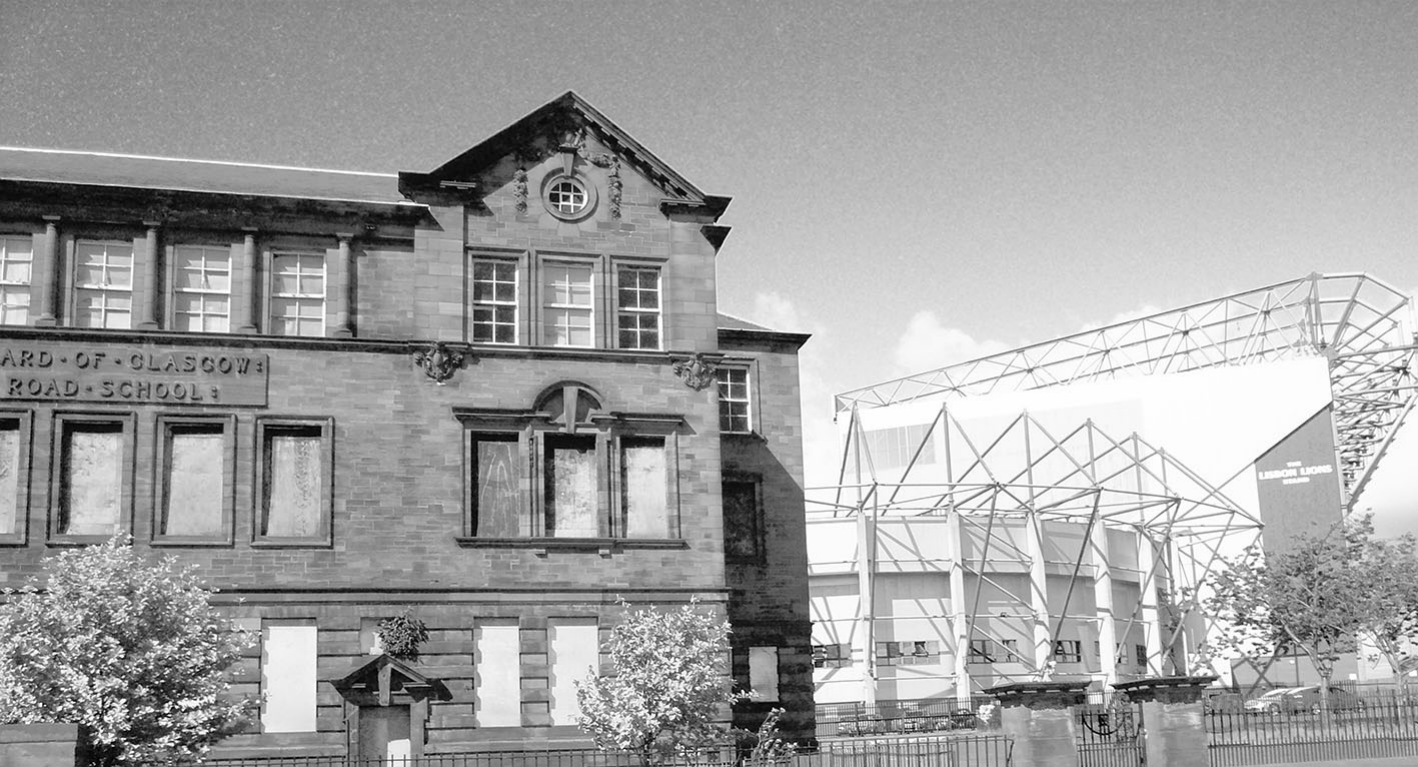
Leisure category: Parkhead Public School

The fourth part of this process was designed to use visual stimuli to elicit the articulation of attachment to place in the context of a changing urban neighbourhood. Lynch’s study of American cities found that following “widespread upheaval” there was an almost “pathological attachment” to the remaining built environment (1960, p. 42). Furthermore, Miller (2003) argues that the meanings that people attach to physical dimensions of places “tend to remain implicit and unexpressed … so long as there is no suggestion of change, no perception of threat” (2003, p. 209). Any disruption to place is therefore often visually evident as buildings/spaces either fall into disrepair, are demolished or are constructed (Brown and Perkins 1992; Devine-Wright 2009). Additionally, existing research has found that these changes often provoke reactions, be it in the form of emotional responses (Fullilove 2004) or through pro-environmental behaviour (Carrus et al. 2014). The photographs were thus designed to engage with this notion of place disruption by highlighting the current condition of the spaces in the context of historic and planned changes to the case study area (Figs. 1, 2, 3 and 4).

The Parkhead neighbourhood of Glasgow, a white working class neighbourhood in the east end of the city, was selected both because it has been the location for successive waves of redevelopment since the deindustrialisation and depopulation of the area in the 1970s, and also part of the site for the newly built facilities for the Commonwealth Games, 2014. Additionally, some existing facilities were also upgraded—principally the major football stadium (Celtic Park) and the local municipal sports which was transformed into the Aquatics Centre for the Games (see Table 2). The focus groups with residents took place before the Commonwealth Games and thus whilst the development of these formal sporting spaces was underway. There was also another form of ongoing neighbourhood change. The informal spaces for sporting activity such as backcourts, streets and vacant land were changed through either housing developments or alterations to the way the area was being policed. These spaces were fluid and subject to ongoing development due to the way in which they were policed and managed and as such are not included in Table 2. Finally, as the research findings show the closure of a local community-based sports complex (Helenvale) approximately 15 years previously was a historic change with which Parkhead residents were still coming to terms with. It seemed plausible, therefore, that the fusion of historical and ongoing changes would be experienced as disruptive by residents—and that this perception might provoke the articulation of any latent attachment to existing recreation-orientated spaces and facilities in the neighbourhood. Photo-elicitation methods were thus utilised in order to visually stimulate the sense of disruption and change to try and unlock the articulation of latent meanings.

**Table 2:** Summary of changes to formal sporting spaces within Parkhead

Facility	Change
National Indoor Sports Arena	Newly constructed for the Commonwealth Games
Velodrome	Newly constructed for the Commonwealth Games
Athletes Village	Newly constructed for the Commonwealth Games
Celtic Park	Upgraded
Municipal Leisure Centre (Tollcross)	Upgraded
Helenvale	Derelict

Crucially, participants were given no indication prior to, or during, the focus group of the research team’s particular interest in sporting recreational spaces. Discussions ranged over attachments to a variety of facets of the built environment such as housing, historic buildings and shops. The focus group schedule did not have specific prompts on attachment to recreational spaces, although two of the fifteen photographs were of such spaces—Celtic Park and the now derelict community sports complex. Each focus group was audio recorded and transcribed; all respondents were anonymised and are referred to in this paper by pseudonym. The transcripts were thematically analysed to explore both the reasons for place attachment and the emotional reactions provoked by changing recreational spaces that were conveyed by participants.

## Attachment to recreational spaces: evidence from the research

The focus groups revealed a range of attachments to sporting recreational spaces, and were volunteered by participants unprompted. Of the spaces identified in Table 2, Helenvale, Tollcross, Celtic Park and the Velodrome were consistently discussed by the participants. Of these neither Tollcross nor the Velodrome had a photograph associated with them yet were volunteered by the participants at various stages of the focus groups. For example, in one focus group, the very first response to the open question ‘what is good about this neighbourhood?’ came from a participant who talked warmly of municipal leisure centre at Tollcross: “it’s got everything there… it’s well used by all ages” (Barry, FG_Older). With regards to the informal spaces then streets, backcourts or wasteland spaces in which football or rounders could take place were also consistently discussed without visual stimuli. Two of the spaces continually volunteered were however the subject of photographs: the derelict community sports complex (Helenvale) and Celtic Park football stadium. Indeed, one participant indicated that he had actually desired that a photograph of the Helenvale complex would be included: “when you said you were doing photos I hoped it would be there” (Danny, FG_Mixed). This suggested that discussion of the complex was part of his personal agenda in relation to participation in the research. Similarly, a different participant surreptitiously took home the photograph of Celtic Park. The fact that the four recreational spaces were offered both as a result of, and also independent of, photo-elicitation reveals that these spaces, for some of the participants, existed in the imagination prior to any visual stimuli being offered.

Moreover, a consistent narrative was evident across the focus groups. This highlighted a sense of cumulative loss of recreational spaces and comprised two main elements: the continual reduction of the land which participants felt was available for them to watch and play sports; and the physical displacement of people within recreational spaces.

That participants experienced a reduction in the land that could be used for recreation was demonstrated across the focus groups. The impact of tenement rehabilitation on the capacity for sport was commented on in this discussion:

Maud: “you know, these old tenements used to have back courts but now it’s car parks

Joan: there isn’t a lot of places that the weans can play

Jan: aye…cause sometimes (…) if they fall and slip they end up

Catherine: exactly getting’, impaled or something (…) like Dracula (laughs)” (FG_Older).

Other examples of loss included the inability to use the swimming pool in Tollcross Leisure Centre whilst preparations were made for the Commonwealth Games, the complete closure of Helenvale, the community sports complex, and the increasingly punitive police presence in the area that prevented young people from playing group games. The identification of reduction in recreational spaces and opportunities to play sport was often set in the context of the sense of dislocation the participants felt.

The discussions suggested that the loss of land was also resulting in the physical displacement of people. Thus it was not just that open spaces had become car parks, but the concomitant increase in traffic in residential neighbourhoods and the fact that both moving and stationary vehicles physically intruded into the spaces available for sporting activities which were also viewed as problematic. There was also a sense that sporting activity was being deliberately pushed out from such spaces both by police officers and by some other residents—“neighbours phone the police for kids having a game, a kick about” (Clare, FG_Mixed) In addition, the redesign of the tenemental built form as a result of new housing developments in the area “did away with” the backcourt spaces popular for such an activity.

In one example, a participant located an historic story of change within a narrative of contemporary change. She described how the spaces used for informal rounders matches in her youth had been lost when a part of the neighbourhood was “flattened” 20 years ago. However, bringing the displacement narrative up-to-date, she indicated: “where it was, [it’s] where they’re building the Velodrome now” (Joan, FG_Older). More striking perhaps were examples of other forms of inappropriate intervention which posed a threat to “community spirit”. Furthermore, Derek described how the capacity to play football in an open space near his home had been undermined by the interventions of local council officers who “cut the trees down that we used to use as goals”. In addition, now when young people build their own goals “the council comes… and breaks them and takes them away.” A final example from the same group related to policing practices towards young people. These were deemed inappropriate, particularly what was described as “harassment” of ordinary activities such as street football and hanging out (FG_Active). However, the introduction of football matches organised by the police as part of a diversionary strategy to deal with gang related activity, appeared to both undermine informal sports activity among non-gang members, and indeed to encourage gang membership. Thus, it was said, the police had taken over street football to the extent that young people could only “get a game” if they were a member of a gang. The themes of loss of previously vibrant spaces, the inextricable connection between time and space, and the physical and social dimensions of sporting spaces recurred throughout the focus groups and were used to help frame the thematic analysis, the findings of which are detailed below.

### Collective memory

**Fig. 5:**
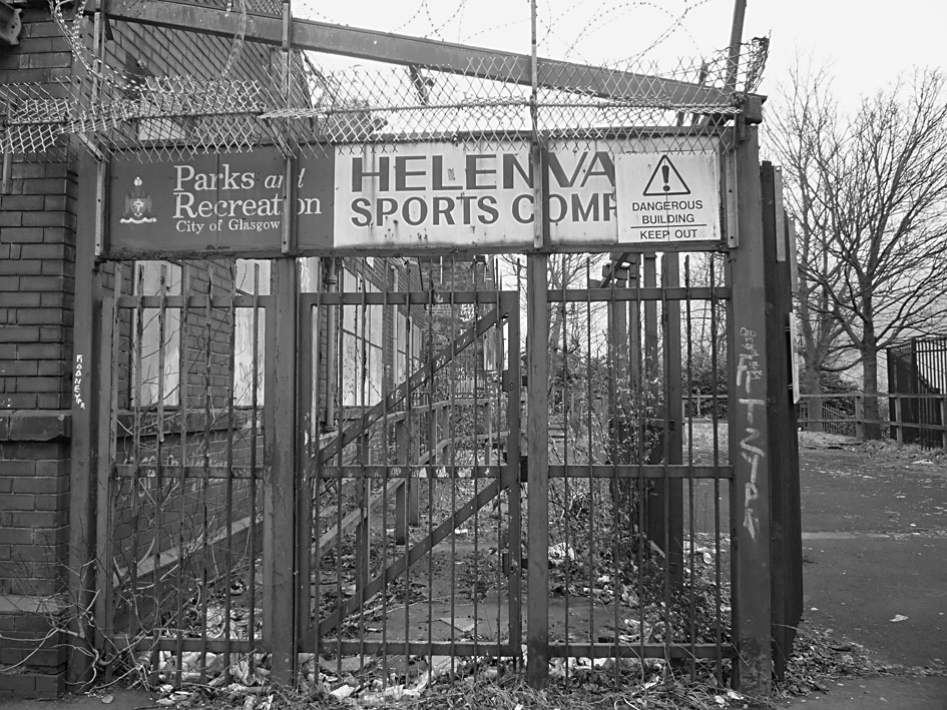
Photograph of the now derelict Helenvale Sports Complex that was shown to participants

Attachments to places in Parkhead were heavily influenced by the memory of former recreational spaces. This was particularly the case in relation to Helenvale, the derelict community sports complex, where individual and collective memory appeared as strong drivers of attachment. Indeed even when the discussion centred on the contemporary photograph (see Fig. 5) which showed it in its derelict state, strong memories surfaced of what the complex had once been. In the mixed-age group, Danny is joined by two other participants in re-imagining himself in the complex in its heyday: “I wish I was still there now [Corrin: aye] [Julie: mmm] because that was a great place- [Corrin: aye”]. In the older-persons group, Jack interjected into an ongoing discussion of the complex the reflection that: “I pass that every day but when you see the photo it looks entirely different.” In his mind there was a disjuncture between how he imagined the complex to look, drawing on his memory of its previous condition juxtaposed with its current condition.

The historic condition of Helenvale, which had been in Parkhead since 1924, and the meaning it held for residents emerged strongly as a shared memory and, indeed, as a common history amongst focus group participants. In each focus group, similar stories were articulated of the complex’s heyday and of how it was first shut down and then abandoned. It was also evident that this history was shared regularly beyond the research setting in ordinary social interactions. In two of the focus groups, participants related how stories of the complex were ‘passed on’ to residents too young to have known it before it was closed down. Christine related how she had taken her older daughter to activities there, but that it had closed before she could do the same with her younger daughter. However, whenever Christine passed the complex with this younger daughter, the daughter would ask her to recount the stories she had clearly already heard about her mother and older sister’s activities in the complex. In a similar vein, Jennifer, a member of the young-persons group, told how her aunt had also passed on stories of the complex to her: “she told me it was pure good, it was the football pitches, tennis, badminton, different clubs, and she still would go there but it’s been shut down and she just never had anywhere else to go”. These collective and received memories formed an integral part of the ‘history’ of Parkhead as bonds were formed between people, through generations and also between people and the recreational spaces.

### Social bonding

In all four focus groups, the now derelict Helenvale complex was considered as having once been the social glue of the neighbourhood. This was expressed in three distinct ways. First, across the focus groups the complex was emphasised as a space of inclusivity especially in the light of the different generations who went to watch events there. This, together with the inter-generational transfer of stories highlighted in Sect. 4.1, suggests the continual presence of Helenvale provided a stimulus for inter-generational relationships. Second, the role of Helenvale in healing rifts was also stressed to different degrees in the focus groups. For example, James (FG_Mixed) stated that: “Parkhead has always been known for… territorialism… this (complex) broke it down a lot because we had teams from Carntyne, Shettleston, all the different areas”. Nowhere is this rift more obvious than between Glasgow’s rival football teams, Celtic and Rangers. However James and others suggested that matches at Helenvale provided an opportunity for fans of both teams to watch the football together: “we had fantastic crowd—we had bigger crowds there than you get at some second division games in Scotland”. Finally, the complex also brought together those who were not natural sports fans. Ruth, for example, recalled that her “dad never ever bothered with football, but he used to go there and watch it, because it was a lot of, you know, local people”.

The focus groups also revealed a connection between other sporting spaces, both formal and informal, and social bonding. This was demonstrated by the perceived place of Celtic Park as integral to the social life of the neighbourhood. The stadium was seen to give the area its ‘identity’ and far from being seen as removed from the daily rituals and rhythms of neighbourhood life, the stadium played a central role. This was demonstrated as it was praised for hosting a variety of community activities and work with schools “yeah my boy’s class learned Japanese in it, and I think most of the kids this year are learning French in it” (Joan, FG_Active). Furthermore the stadium was also praised for generating income for the area and for being a visual landmark. The place of the stadium in generating social bonds was summarised by Linda (FG_Active) as “a place where people go as a family and it keeps that kind of family together in the kind of community spirit and everybody goes there because they’re passionate about something…”.

This sense of a community spirit and social bonding was also indicated by Danny with reference to informal street spaces where:

(We) used to go out and play football there, everybody in the summer, just everybody that stayed in their houses, the dads and everything, and the mums would sit together and get steaming and we would just run about playing football (laughter) it was brilliant, I loved it man.

The findings demonstrate both the existence of attachments to recreational spaces but also the different drivers for this attachment. The temporal, spatial and social were inextricably connected in these narratives of recreational spaces. Crucially, these spaces were never discussed as static but were always considered as part of a moving continuum whether that was looking back in time or projecting forward. Neither were they discussed in an emotional vacuum, but were suffused with layers of meaning which were unlocked through the use of this temporal continuum. More specifically, the temporal/spatial nexus revealed that the capacity for social bonds to form or be maintained is undermined by cumulative disruptions to these sites such as closure, dereliction or restriction of access.

### Emotional responses

The thematic analysis of the focus groups revealed recurring emotional responses across the four focus groups. This section explores the range of both positive and negative emotions provoked by changes to recreational spaces that were also identified by Bale (2000) and is split into two sections: positive and negative in which pride, sadness, fear and anger are articulated. In doing so it is not the intention to present these as opposites but to recognise the complexity of emotional attachments and to identify and explain the context for their existence. Moreover, the section seeks to understand how a spectrum of emotion can be generated through developing an understanding how people’s attachments to recreational spaces are formed.

#### Positivity: pride

The temporal dimension to place attachment was again in evidence as participants demonstrated their pride in the historical aspects of Parkhead’s sporting activity. This was often recalled through the succession of places, events and people that gave Parkhead its sporting reputation. John spoke with pride when remembering that Helenvale “was about the… first… Astroturf park in Scotland, “and it was up there and we had a fantastic league” (John, FG_Mixed). This was supported by the pride the participants felt in explaining to people where they lived: “aye because we went away last year and we said where we were from and we (said we) stayed (lived) near Celtic Park … it’s automatically: ‘Celtic Park’” (June, FG_Mixed).

The neighbourhood’s connection to famous sporting heroes was also demonstrated across the focus groups. John demonstrated his further pride for Helenvale by stating the iconic Scottish footballer Tommy Burns had played there. In addition, June stated that Parkhead was the home of the famous Lisbon Lions and that all but one member of the squad “that won the European Cup” was born within ten miles of the stadium. Pride was also demonstrated towards friends who had played football in the neighbourhood especially one who had recently signed a professional contract with a Scottish Premier League team. This also translated into aspiration as the focus group discussed the stigma attached to the East End: “I don’t know if it makes a difference but what she’s saying is anybody from the East End can do it (make a living out of being a professional footballer)” (Danny, FG_Mixed). Here the temporal and spatial nexus played out as pride in the previous sporting achievements of the area and was used to ground a positive view for the future. The pride participants felt for sporting achievements in Parkhead were important factors in their attachment to, and identification with, certain spaces within the neighbourhood. However, the emotional reactions towards recreational spaces were not always positive as the perceived deterioration of these once proud places provoked anger, fear and sadness in the participants.

#### Negativity: sadness, anger, fear

The pride in the neighbourhood’s sporting achievements demonstrated an aspect of the ‘dynamic tension…between positive and negative affect’ (Manzo 2014). In line with the original thesis surrounding the threat associated with place disruption, it was clear that cumulative changes to Parkhead provoked negative emotions. Dominant within these were sadness and anger at the loss of familiar recreational spaces, but also fear as to what the next stage of redevelopment for the Commonwealth Games would mean for local people and their recreational spaces. Helenvale was again a source of emotion.

The photograph provoked strong language and an intense emotional response in the older-persons group:

Researcher: “so looking at it now what do you think of it now?

Maureen: what do I think? It’s a - I was going to swear there [Christine: It’s an eyesore isn’t] - it’s a bloody disgrace

Enid: just left it lying

Researcher: other [reactions]?

Jacqui: Are we allowed to swear in here?

Maureen: bloody disgrace”.

Wayne supported this view by stating that the closure of Helenvale was “one of the biggest crimes… absolutely unbelievable shutting that down” (FG_Active). Other participants felt it was “ridiculous” (John, FG_Mixed) and a “sin” (Caroline, FG_Older) to leave the building to deteriorate and thus built on Wayne’s use of an emotionally-laden vocabulary. There was a similar story with the informal street spaces as participants lamented the loss of the impromptu games in streets and on wastelands:

Corrin: “see kids can’t get playing football… it’s the same with where I stay (live),” (FG_Active).

Danny: “I wish it was still there now”

Clare: “aye” (FG_Mixed).

This sadness, provoked by recalling the loss of so many different kinds of sporting recreational spaces, was also used to express fears concerning the next phase of redevelopment associated with the Commonwealth Games. Janet stated that she didn’t think they’d “ever get back the spirit they had in there (Helenvale)” (FG_Mixed). Furthermore, the fear surrounding these new facilities was also demonstrated in terms of social exclusion. In the mixed-age group, Danny asked about the new Velodrome:

plus, what’s going to happen after the Commonwealth Games are away, we’re going to be left with it, what’s going to happen then… is it going to get used and is it going to cost us more or is it going to cost everybody in Parkhead more…?

In addition fears surrounding the level of access to existing sporting spaces were also demonstrated. One participant was concerned that the transformation of Tollcross Leisure Centre into a “world class pool” for the Aquatics events at the Commonwealth Games meant the outsiders were “bussed in from private schools”, thus turning “locals away” (Danny, FG_Mixed). This concern was shared across the age spectrum as Chelsea from the young-persons group stated:

the prices might be quite too high… because like, there is like a lot a poverty and if they’re bringing the Velodrome in, people who want to use won’t be able to, because they’ll still be paying money to get into it… it’s not really going to be good, because there (is) still poverty in the streets. So the government isn’t really doing anything for it.

This fear surrounding access to local recreational spaces arose from a sense of the cumulative changes to the facilities in Parkhead. The participants moved back and forward in time to base their arguments on the received memories of the area to inform their sense of the future for Parkhead. Again, Helenvale provoked strong reactions as one participant discussed his version of the mistakes that led to the centre’s closure:

“… they brought American football in, it had become the latest fad, and they brought that in. And it was teams from the west end and things like that… And they just put prices right sky high … And when American football, when the fad finished, and that went away, people couldn’t afford it anymore and it was left to go to, eh, ruin, and it’s just been left there for years” (John, FG_Mixed).

Such concerns align with Danny’s fears indicated already (“what’s going to happen after the Commonwealth Games are away (finished)?”) and suggest that collective memory of past redevelopments may form the basis of fears about the future of recreational spaces. Moreover, concerns about the affordability of the new sporting venues voiced by participants and noted earlier also appear to find a parallel with Helenvale. The “sky high” prices that accompanied the change of sport from football to American football are argued to have destroyed the capacity of locals to use the complex. Moreover, the change was imposed from outside, by some unspecified “they” and was geared to outsiders, to “teams from the (affluent) west end” of the city. Locals felt marginal and excluded as Helenvale changed its use and this fear was then projected into the future. Participants across the four focus groups conveyed their fear that, in upgrading existing spaces and creating new facilities “to suit the Commonwealth Games”, the capacity for locals to access them and thus further build up attachments to them would be undermined.

This section has demonstrated the complexity of emotions expressed by participants. Pride, sadness, fear and anger co-existed within the participants’ emotional register as the spaces for recreation were consistently disrupted. These reactions were grounded in both the reality of historic and cumulative physical change but also existed in the ways in which the participants imagined future changes to the ways in which local people would be able to access the new spaces as the neighbourhood suffered from cumulative disruption.

## Conclusion

This paper has examined how the physical and social dimension of sporting recreational spaces influenced the formation and expression of attachments. The research has highlighted the complexity of attachments and in doing so engages with a desire to analyse the “inherently dynamic nature of place attachment”. It does so in three main ways.

First, it has illustrated the constant interplay between the temporal and spatial. In particular the ways in which historic and planned changes to recreational sporting spaces provoked emotional reactions amongst the participants was revealed. This was apparent with the recalled spaces of the past, the felt spaces of the present, and the imagined spaces of the future. A key narrative throughout the case study was the role of cumulative memories and experiences in neighbourhoods and the ways in which successive redevelopments altered the ways in which people felt, used and experienced recreational spaces, and thus the strength of attachments to place. Successive top-down urban strategies, in particular, the changes to the tenement style of living in Parkhead, the increased policing of the area, and the redevelopment associated with the Commonwealth Games had profound consequences for the availability of recreational spaces. The reality of the continuing physical loss of sporting spaces co-existed with the fear of losing future access to new sporting spaces.

Second, the inextricable connection between physical spaces and social networks within place attachment was demonstrated through the findings. Memories, meanings and projections were often intimately connected to the physical look and feel of spaces. As such the derelict state of Helenvale was mourned as much as the iconic design of the new Velodrome was feared. The loss of recreational spaces and the prohibitive costs of the new buildings were lamented for restricting opportunities for recreation but also for the perceived reduction in social bonding, sometimes across generations, that resulted from interaction in these spaces. The focus on sporting recreational spaces therefore offers an important insight into the extent to which physical spaces are not mere backdrops for social action but are active agents in shaping the behavioural practices of the residents of urban neighbourhoods. Disruption to these physical spaces unlocked a set of emotional reactions that were fundamentally provoked either by the memory of sporting spaces, or by their current condition, or by the imagined future of physical spaces. At all times the visibility of place, stimulated by the photographs, provoked a series of complex and layered embodied reactions. In this way the photo-elicitation methodology worked to reveal this relationship between the physical and the social. The photos allowed the research team to access “the embodied connections that people have with particular places” (Jones and Evans 2012, p. 316) as they became a mnemonic that helped to stimulate both the rhetoric of remembrance and the reality of physical change. The findings from the focus groups also aligned with findings in the literature review, most notably from Bale (2000), Stedman (2003), Stone (2007) and Wood and Gabie (2011) to demonstrate the ways in which attachment to recreational spaces was bound up with a complex set of social relations in which the physical structures provided a place in which people could interact and thus develop a range of lived experiences which in turn provided layers of meanings and memories.

Finally, the photographs also provoked emotional responses to cumulative physical changes in the neighbourhood. These responses encompassed both a broad spectrum ranging from pride to anger but intriguingly, despite the superficial binary opposites of ‘positive’ and ‘negative’, also demonstrated the co-existence of these feelings. This aligns with the recent ‘emotional turn’ (Stets and Turner 2014; Wetherall 2012) in the social sciences, and these findings help to demonstrate the value of understanding affective bonds in the context of urban change. This emotional turn has embraced a range of research fields but the place attachment literature has still tended to be “narrowly focussed on *positive* affect and experience” (Manzo 2014, p. 179; emphasis added). As such the more negative ‘shadow side’ of place attachments first identified by Chawla (1992) is now re-emerging as an area of research. Alongside this is recognition that research should probe the ways in which a range of emotions are generated and co-exist in the context of place attachment. Hester (2014) believes that place attachments result from a desire to satisfy basic wishes such as security but that alongside each wish is a competing “monster” such as fear which if not properly understood can “block our awareness of place attachments and how they can inform place making” (p. 203). This is especially important in the context of Parkhead as fears concerning future access to recreational spaces were grounded in anger and sadness about the current dereliction of former recreational spaces. Understanding the affective materiality of urban spaces and how attachments and emotions are stimulated by physical spaces is thus vital for examining the extent to which these embodied connections to the built environment have the “potential to improve the quality of subsequent redevelopments.” (Jones and Evans 2012, p. 2316).

The relationship between the social and physical dimensions of place attachment is an emerging area of the literature and this paper has demonstrated that recreational spaces can provide a vital lens to explore this. Furthermore, it has demonstrated that the embodied connections that stimulate attachments to places, such as memories and stories, are provoked by the visibility and immediacy of physical change. The fluidity of place and of place attachments is evident in this paper. In the case of attachments to Parkhead’s recreational spaces, a relational account of the cumulative layers of temporal and spatial and social and physical changes can unlock the intricate and complex dimensions of place attachment in a neighbourhood undergoing redevelopment.

## References

[CR1] Altman I, Low SM (1992). Place attachment.

[CR2] Armstrong G (1998). Football hooligans: Knowing the score.

[CR3] Bale J (2000). The changing face of football: Stadiums and communities. Soccer & Society.

[CR5] Bricker KS, Kerstetter DH (2000). Level of specialization and place attachment: An exploratory study of whitewater recreationists. Leisure Sciences.

[CR6] Brown B, Perkins D, Altman I, Low SM (1992). Disruptions in place attachment. Place attachment.

[CR7] Carrus G, Manzo L, Devine-Wright P (2014). Place attachment, community identification, and pro-environment behaviour. Place attachment: Advances in theory, methods and applications.

[CR8] Chawla L, Altman I, Low SM (1992). Childhood place attachments. Place attachment.

[CR9] Collier J, Collier M (1986). Visual anthropology: Photography as a research method.

[CR10] Devine-Wright P (2009). Rethinking NIMBYism: The role of place attachment and place identity in explaining place-protective action. Journal of Community and Applied Social Psychology.

[CR11] Dixon J, Durrheim K (2000). Displacing place-identity: A discursive approach to locating self and others. British Journal of Social Psychology.

[CR12] Fullilove MT (2004). Root Shock: How tearing up city neighborhoods hurt America and what we can do about it.

[CR13] Hester RT, Manzo L, Devine-Wright P (2014). Do not detach! Instructions from and for community design. Place attachment: Advances in theory, methods and applications.

[CR14] Hillier J, Rooksby E (2005). Habitus: A sense of place.

[CR15] Hinch T, Higham J (2011). Sport tourism development.

[CR16] Jones P, Evans J (2012). Rescue geography: place making, affect and regeneration. Urban Studies.

[CR17] Kyle G, Graefe A, Manning R, Bacon J (2003). An examination of the relationship between leisure activity involvement and place attachment among hikers along the Appalachian Trail. Journal of Leisure Research.

[CR18] Lewicka M (2010). What makes neighborhood different from home and city? Effects of place scale on place attachment. Journal of Environmental Psychology.

[CR19] Lewicka M (2011). Place attachment: How far have we come in the last 40 years?. Journal of Environmental Psychology.

[CR20] Light R (2013). Ordinary working men…transformed into giants on the rugby field’: Individual and collective memory in oral histories of Rugby League. International Journal of the History of Sport.

[CR21] Livingston M, Bailey N, Kearns A (2010). Neighbourhood attachment in deprived areas: Evidence from the north of England. Journal of Housing and the Built Environment.

[CR22] Lynch K (1960). The image of the city.

[CR23] Manzo LC (2003). Beyond house and haven: Toward a revisioning of emotional relationships with places. Journal of Environmental Psychology.

[CR24] Manzo LC, Manzo LC, Devine-Wright P (2014). Exploring the shadow side: Place attachment in the context of stigma, displacement and social housing. Place Attachment: Advances in theory, methods and applications.

[CR39] Manzo, L. C., & Devine-Wright P. (Eds.), (2014). *Place Attachment: Advances in theory, methods and applications*. Abingdon: Routledge

[CR25] Miller MJ (2003). The representation of place, urban planning and protest in France and Great Britain, 1950–1980.

[CR26] Proshansky HM, Fabian AK, Kaminoff R (1983). Place-identity: Physical World Socialization of the Self. Journal of Environmental Psychology.

[CR27] Ramshaw G, Gammon S (2010). On home ground? Twickenham Stadium Tours and the construction of sport heritage. Journal of Heritage Tourism.

[CR28] Ramshaw G, Hinch T (2006). Place identity and sport tourism: The case of the Heritage Classic ice hockey event. Current Issues in Tourism.

[CR29] Robson G (2000). No one likes us, we don’t care’: The myth and reality of Millwall fandom.

[CR30] Scottish Government (2010). Designing streets.

[CR31] Scottish Government (2014). Our place in time: The historic environment strategy for Scotland.

[CR32] Stedman RC (2003). Is it really just a social construction?: The contribution of the physical environment to sense of place. Society and Natural Resources.

[CR33] Stedman RC, Manzo L, Devine-Wright P (2014). Photo-based methods for understanding place meanings as foundations of attachment. Place attachment: advances in theory, methods and applications.

[CR34] Stets JE, Turner J (2014). Handbook of the sociology of emotions.

[CR35] Stone C (2007). The role of football in everyday life. Soccer & Society.

[CR36] Tuan Yi-Fu (1977). Space and place: The perspective of experience.

[CR37] Wetherall M (2012). Affect and emotion: A new social science understanding.

[CR38] Wood J, Gabie N (2011). The football ground and visual culture: recapturing place, memory and meaning at Ayresome Park. International Journal of the History of Sport.

